# Plasma proteomic profiling in postural orthostatic tachycardia syndrome (POTS) reveals new disease pathways

**DOI:** 10.1038/s41598-022-24729-x

**Published:** 2022-11-21

**Authors:** Madeleine Johansson, Hong Yan, Charlotte Welinder, Ákos Végvári, Viktor Hamrefors, Magnus Bäck, Richard Sutton, Artur Fedorowski

**Affiliations:** 1 Clinical Research Center, Department of Clinical Sciences-Hypertension and Cardiovascular Disease, Faculty of Medicine, Lund University, Box 50332, 202 13 Malmö, Sweden; 2 Department of Cardiology, Skåne University Hospital, Malmö, Sweden; 3 Swedish National Infrastructure for Biological Mass Spectrometry, BioMS, Lund, Sweden; 4 Department of Medical Biochemistry and Biophysics, Karolinska Institutet, Stockholm, Sweden; 5 Department of Cardiology, Karolinska University Hospital, Stockholm, Sweden; 6 Department of Medicine, Karolinska Institutet, Stockholm, Sweden; 7 National Heart and Lung Institute, Department of Cardiology, Hammersmith Hospital Campus of Imperial College, London, UK

**Keywords:** Biomarkers, Biomarkers, Cardiology, Medical research

## Abstract

Postural orthostatic tachycardia syndrome (POTS) is a cardiovascular autonomic disorder characterized by excessive heart rate increase on standing, leading to debilitating symptoms with limited therapeutic possibilities. Proteomics is a large-scale study of proteins that enables a systematic unbiased view on disease and health, allowing stratification of patients based on their protein background. The aim of the present study was to determine plasma protein biomarkers of POTS and to reveal proteomic pathways differentially regulated in POTS. We performed an age- and sex-matched, case–control study in 130 individuals (case–control ratio 1:1) including POTS and healthy controls. Mean age in POTS was 30 ± 9.8 years (84.6% women) versus controls 31 ± 9.8 years (80.0% women). We analyzed plasma proteins using data-independent acquisition (DIA) mass spectrometry. Pathway analysis of significantly differently expressed proteins was executed using a cutoff log2 fold change set to 1.2 and false discovery rate (p-value) of < 0.05. A total of 393 differential plasma proteins were identified. Label-free quantification of DIA-data identified 30 differentially expressed proteins in POTS compared with healthy controls. Pathway analysis identified the strongest network interactions particularly for proteins involved in thrombogenicity and enhanced platelet activity, but also inflammation, cardiac contractility and hypertrophy, and increased adrenergic activity. Our observations generated by the first use a label-free unbiased quantification reveal the proteomic footprint of POTS in terms of a hypercoagulable state, proinflammatory state, enhanced cardiac contractility and hypertrophy, skeletal muscle expression, and adrenergic activity. These findings support the hypothesis that POTS may be an autoimmune, inflammatory and hyperadrenergic disorder.

## Introduction

Postural orthostatic tachycardia syndrome (POTS) is a cardiovascular autonomic disorder characterized by an excessive heart rate increase of > 30 beats per minute (bpm) or a heart rate of > 120 bpm and symptoms of orthostatic intolerance when assuming upright posture^1^. The syndrome affects predominantly young women (70–80%) within a range of 15–40 years old. It is estimated that POTS affects approximately 3 million people in the United States^2^, and since the start of COVID-19 pandemic it has been observed that individuals may also develop POTS following a SARS-CoV-2 infection^3,4^ which implies an even greater clinical burden.

The heterogenous and multifactorial etiology, poses substantial challenges for physicians striving to provide targeted treatment options for patients, and few studies have identified promising biomarkers^5,6^. A greater mechanistic understanding and clinical phenotyping, as well as detection of novel POTS biomarkers are required to gauge disease activity, severity and response to therapy.

Data independent acquisition (DIA) is a mass spectrometric technique, also known as sequential window acquisition of all theoretical mass spectra (SWATH-MS), offering a powerful proteomic approach for unbiased discovery, generating comprehensive, digital proteome maps and highly reproducible analysis of hundreds of proteins in the plasma^7^. SWATH is a specific variant of DIA technology, coupled with peptide spectral library match enables precise label-free quantification of the proteome^8^. Compared with conventional mass spectrometry, DIA is based on the fragmentation of all precursor ions identified in a scan rather than only acquiring fragmentation data from a predefined set of selected precursor ions. This improves the depth of sample analysis, reproducibility of the protein identification and both accuracy and precision of the data quantification. In the present study, we aimed to identify the proteomic footprint of POTS versus healthy controls using the unbiased proteomics DIA label-free quantification method.

## Methods

### Ethical approval

The study has been approved by the Regional Ethical Review Board in Lund, Sweden (no 82/2008), with amendment in 2017. All study participants provided informed written consent. All procedures were carried out in line with relevant current guidelines and regulations.

### Study population and design

We performed a sex- and age-matched case–control study, analyzing plasma samples of 130 individuals (case–control ratio 1:1). Sixty-five POTS patients with a sustained heart rate increase of ≥ 30 bpm during head-tilt (HUT) and chronic symptoms for ≥ 6 months without orthostatic hypotension^1,2^, were randomly selected from the Syncope Study of Unselected Population in Malmö (SYSTEMA) cohort (including moderate to severe POTS with maximum delta heart rate at 5 min ranging from 30 to 73 bpm, regardless of history of complete loss of consciousness. SYSTEMA enrolled over 2200 patients investigated for syncope at the Skåne University Hospital in Malmö, Sweden between 2008 and 2020. Details of the SYSTEMA cohort are described elsewhere^9^. Only participants with available plasma samples were included.

Sixty-five healthy age- and sex-matched controls (without CVD, diabetes autoimmune disease, immunosuppressive treatment, history of cancer or other proliferative disease, and psychiatric disorder) were included. Healthy controls were recruited through personal invitation, among healthy medical students, hospital staff and younger participants of parallel population-based epidemiological programs in Malmö, Sweden. No financial incentive was given. Controls completed a self-administered questionnaire regarding their past and current medical history, and were examined with ECG, blood pressure measurements and active standing test at the Clinical Research Unit of Dept. of Internal Medicine, Skåne University Hospital, Malmö, Sweden. Controls had no history of syncope, POTS, orthostatic intolerance, cancer, cardiovascular or endocrine disease.

### Investigation protocol

Cases and controls were examined in a dedicated clinical research unit at Skåne University Hospital, Malmö. All cardiovascular pharmacological agents were discontinued 72 h prior to examination. Study participants performed an active standing test, with 10-min rest in the supine position prior to standing. Blood pressure and heart rate were measured twice in the supine position by a validated automated oscillometer device (Omron, Kyoto, Japan), and then after 1, 3, 5, and 10 min of standing. An average of two measurements was used for group comparisons.

### Mass spectrometric analysis

The mass spectrometry proteomics data have been deposited at the ProteomeXchange Consortium via the PRIDE^10^ partner repository with the dataset identifier PXD031458. Details about the plasma sample preparation can be found in the Supplement. Twenty µL of each sample were loaded into Evosep tip for the LC–MS/MS analysis^11^. The plasma samples were analyzed on a timsTOF Pro mass spectrometer (Bruker Daltonik, Germany) coupled with Evosep One (Evosep, Odense, Denmark) with 60 samples per day (SPD) and DIA-PASEF long gradient method. Solvent A (0.1% FA in water) and solvent B (0.1% FA in ACN) were used to create gradient and the analytical column, EV1109 (150 μm × 8 cm, 1.5 µm) used for the sample analysis. Details on data analysis using DIA method is outlined in the Supplement.

### Protein pathway analysis

Protein pathways were analyzed using the bioinformatic tool STRING (Search Tool for Retrieval of Interacting Genes/Proteins) to acquire protein–protein interaction (PPI) networks^12^. The STRING database aims to integrate all known and predicted associations between proteins, including both physical interactions as well as functional associations. The significantly up- or down-regulated proteins were submitted separately to full STRING network search with medium interaction score (0.4) and a false discovery rate (FDR) ≤ 0.05 was used when classifying the cellular component (Gene Ontology) of each protein. For each obtained network, PPI enrichment p-value was reported. p-values are corrected for multiple testing within each category using the Benjamini–Hochberg procedure.

### Statistical analyses

Continuous data are shown as mean ± standard deviation (all variables were normally distributed), whereas frequencies are used to describe categorical data. Continuous variables were compared using the paired Student’s *t* test for matched couples. Paired and multiple proportions were compared using Pearson’s chi-square test.

DIA data were analyzed using Spectronaut (version 15, Biognosys, Switzerland) using the directDIA workflow. The data was based on extracted maximum intensities of both precursors and fragment ions. Default settings of the Spectronaut method were applied for both identification and quantification of peptides and proteins. Data was filtered by q-value and an automatic strategy was used for the cross-run normalization. The protein quantitative report was exported from Spectronaut and downstream statistical analysis was performed with Perseus 1.6.14.0^13^. Label-free quantification (LFQ) of DIA data was log2-transformed and normalized to median value of each sample.

Statistical analyses were performed in IBM SPSS Statistics 27 (IBM Corporation, Armonk, NY, USA), two-sample test (unpaired Student’s *t* test) was performed for the proteomics data within the Perseus Software, and data were plotted with R Studio (Boston, MA, USA).

## Results

Study characteristics of the population are shown in Table 1. The mean age of POTS and healthy controls were 30 ± 9.8 years (84.6% women) and 31 ± 9.8 years (80.0% women), receptively. Supine heart rate (70.5 ± 11.6 vs. 62.9 ± 10.1 bpm, p < 0.001) and heart rate after 5 min of standing were significantly higher in POTS versus healthy controls (108.7 ± 17.4 bpm vs. 82.7 ± 12.7 bpm, p < 0.001).

**Table 1:** Study characteristics of the population.

Characteristics	POTS (n = 65)	Healthy controls (n = 65)	p-value
Mean age, years ± SD (age range)	30 ± 9.8 (17–61)	31 ± 9.8 (16–62)	0.37
Female, n (%)	55 (84.6)	52 (80.0)	0.49
Systolic BP supine, mmHg ± SD	116.1 ± 12.6	112.8 ± 10.3	0.11
Diastolic BP supine, mmHg ± SD	71.7 ± 8.8	67.9 ± 7.9	0.01
Heart rate supine, bpm ± SD	70.5 ± 11.6	62.9 ± 10.1	< 0.001
Heart rate 5 min standing, bpm ± SD	108.7 ± 17.4	82.7 ± 12.7	< 0.001

*BP* blood pressure, *bpm* beats per minute, *POTS* postural orthostatic tachycardia syndrome, *SD* standard deviation.

### Explorative proteomic analysis

**Figure 1:**
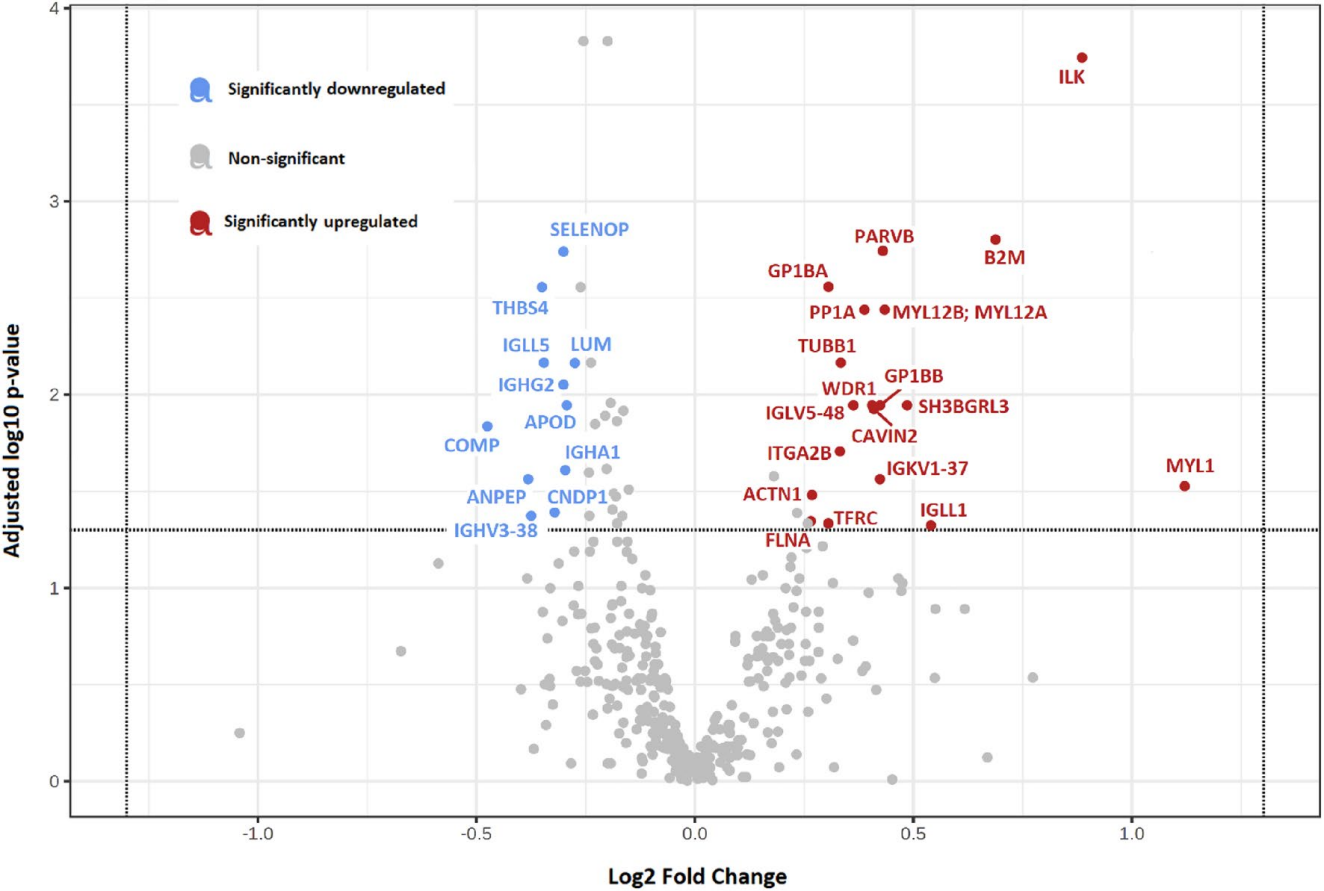
Proteomic footprint of significantly dysregulated proteins in POTS. Volcano plot illustrating 30 significantly dysregulated proteins in POTS. Eleven downregulated proteins in blue and 19 upregulated proteins in red. Complete names of proteins are found in Table 2.

In the explorative analysis, a total of 393 unbiased plasma proteins were detected. The complete list of proteins can be found in the Supplementary Table S1. Overall, label-free protein quantification identified proteins significantly differently expressed in POTS, 19 upregulated and 11 downregulated proteins (Fig. 1, Table 2) using a cutoff fold change set to 1.2 and FDR (q-value) of < 0.05.

**Table 2:** Significantly dysregulated proteins in POTS identified using data-independent acquisition (DIA) label-free quantification mass spectrometry.

Protein name	Gene name	Fold change	Log2 Fold change	p-value
**Upregulated proteins**				
Alpha-actinin-1	ACTN1	1.20	0.27	0.003
**Beta-2-microglobulin**	**B2M**	**1.61**	**0.69**	**< 0.001**
**Beta-parvin**	**PARVB**	**1.35**	**0.43**	**< 0.001**
Caveolae-associated protein 2	CAVIN2	1.33	0.41	0.001
**Filamin-A**	**FLNA**	**1.20**	**0.27**	**0.006**
Immunoglobulin lambda-like polypeptide 1	IGLL1	1.45	0.54	0.006
Integrin alpha-IIb	ITGA2B	1.26	0.33	0.001
**Integrin-linked protein kinase**	**ILK**	**1.85**	**0.89**	**< 0.001**
**Myosin light chain 1/3, skeletal muscle isoform**	**MYL1**	**2.17**	**1.12**	**0.003**
**Myosin regulatory light chain 12B** **Myosin regulatory light chain 12A**	**MYL12B** **MYL12A**	**1.35**	**0.43**	**< 0.001**
**Peptidyl-prolyl cis–trans isomerase A**	**PP1A**	**1.31**	**0.39**	**< 0.001**
**Platelet glycoprotein Ib alpha chain**	**GP1BA**	**1.23**	**0.30**	**< 0.001**
**Platelet glycoprotein Ib beta chain**	**GP1BB**	**1.34**	**0.42**	**0.001**
Probable non-functional immunoglobulin lambda variable 5–48	IGLV5-48	1.29	0.36	0.001
Probable non-functional immunoglobulin kappa variable 1–37	IGKV1-37	1.34	0.42	0.002
SH3 domain-binding glutamic acid-rich-like protein 3	SH3BGRL3	1.40	0.49	0.001
Transferrin receptor protein 1	TFRC	1.24	0.31	0.006
**Tubulin beta-1 chain**	**TUBB1**	**1.26**	**0.34**	**< 0.001**
**WD repeat-containing protein 1**	**WDR1**	**1.32**	**0.41**	**0.001**
**Downregulated proteins**				
Aminopeptidase N	ANPEP	0.77	− 0.38	0.002
Apolipoprotein D	APOD	0.82	− 0.29	0.001
Beta-Ala-His dipeptidase	CNDP1	0.80	− 0.32	0.005
**Cartilage oligomeric matrix protein**	**COMP**	**0.72**	− **0.48**	**0.001**
Immunoglobulin heavy constant alpha 1	IGHA1	0.81	− 0.30	0.002
Immunoglobulin heavy constant gamma 2	IGHG2	0.82	− 0.29	< 0.001
Immunoglobulin lambda-like polypeptide 5	IGLL5	0.79	− 0.35	< 0.001
**Lumican**	**LUM**	**0.83**	− **0.26**	**< 0.001**
Probable non-functional immunoglobulin heavy variable 3–38	IGHV3-38	0.77	− 0.37	0.005
Selenoprotein P	SELENOP	0.81	− 0.30	< 0.001
**Thrombospondin-4**	**THBS4**	**0.78**	− **0.35**	**< 0.001**

**Figure 2:**
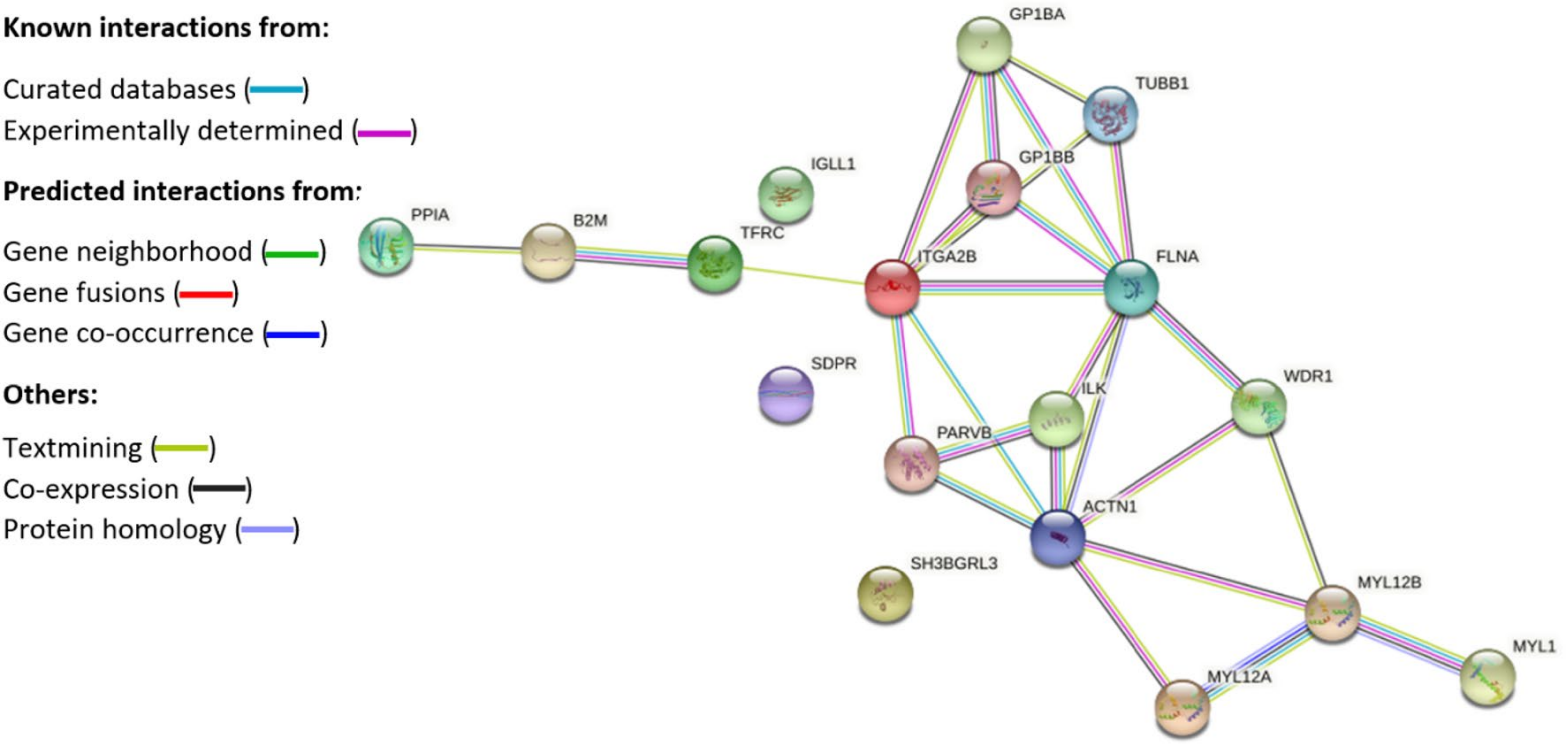
Protein–protein pathway analysis illustrating significantly upregulated proteins in POTS. Protein–protein interaction analysis of 18 out of 19 upregulated proteins with association networks detected by STRING in POTS. In the network figures, each protein is represented by a colored node while protein–protein interaction and association are represented by a line. The strongest network interactions in POTS were particularly associated with a hypercoagulable state and upregulated expression of proteins related to platelet activity, but also enhanced inflammation, cardiac contractility and hypertrophy, skeletal muscle expression, and adrenergic activity. Complete names of proteins are found in Table 2.

**Figure 3:**
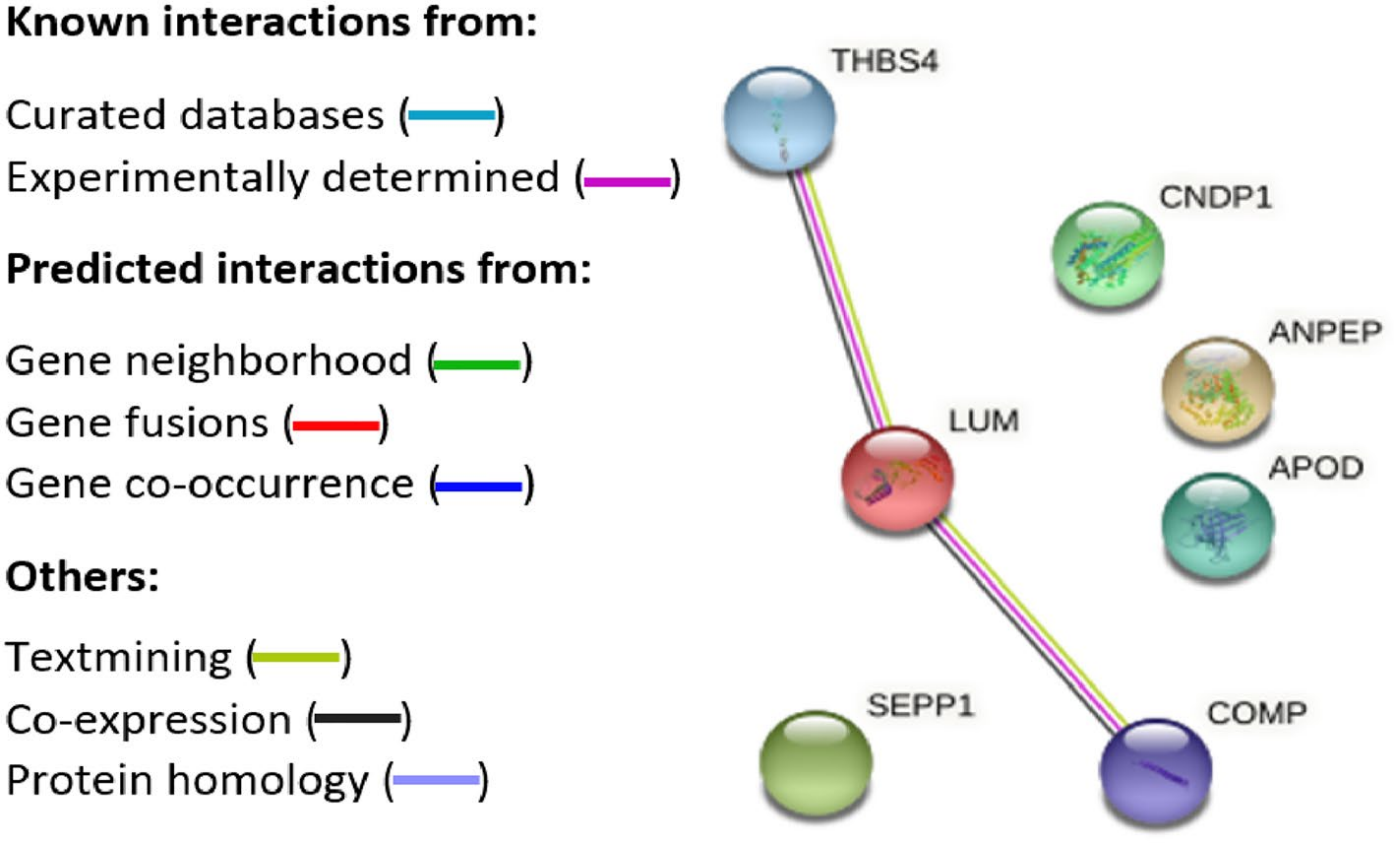
Protein–protein pathway analysis illustrating significantly downregulated proteins in POTS. Protein–protein interaction analysis of 7 out of 11 upregulated proteins with association networks detected by STRING in POTS. In the network figures, each protein is represented by a colored node while protein–protein interaction and association are represented by a line. The strongest network interactions in POTS were particularly associated with a hypercoagulable state, inflammation, and enhanced cardiac contractility and hypertrophy. Complete names of proteins are found in Table 2.

Label-free protein quantification identified 30 significantly dysregulated proteins in POTS, 19 upregulated and 11 downregulated proteins using a cutoff log2 fold change set to 1.2 and false discovery rate (p-value) of < 0.05. Proteins in bold displayed a strong protein–protein interaction in the STRING pathway analysis (Figs. 2, 3).

### Pathway analysis

STRING pathway analysis of proteins provided a network of upregulated (protein–protein interaction (PPI) enrichment p-value: < 1.0e−16, Fig. 2) and downregulated (PPI enrichment p-value: 0.0085, Fig. 3) proteins in POTS. The strongest network interaction among upregulated proteins was particularly found for proteins related to platelet aggregation (FDR 6.88e−06, Supplementary Table S2) and activation (FDR 1.39e−05, Supplementary Table S2). Proteins with significant p-value (< 0.05) without application of fold-change are displayed in the pathway analysis in the Supplementary Fig. 1. As shown, the proteins did not differ much compared with Fig. 2 which identified 19 proteins versus 21 proteins. We also noted network interactions for proteins involved in proinflammatory states, enhanced cardiac contractility and hypertrophy, as well as skeletal muscle expression and adrenergic activity.

## Discussion

In this case–control study, an unbiased DIA proteomic analysis identified 30 plasma proteomic biomarkers that were differentially expressed in postural orthostatic tachycardia syndrome (POTS) compared with healthy, age- and sex-matched controls. In particular, we identified significant up- and downregulated expression of proteins related to platelet activity, cardiac contractility, hypertrophy, proinflammation, skeletal muscle expression, and adrenergic activity.

### Procoagulant changes and enhanced platelet activity in POTS

We observed significant upregulation of platelet-related proteins, such as glycoproteins 1B (GP1BA and GP1BB) which serve as receptors for von Willebrand factor (vWF) and mediate vWF-dependent platelet adhesion to injured vascular surfaces in the arterial circulation, facilitate hemostasis^14^. Also, filamin A (FLNA), which interacts with GP1BA, strengthens adhesion of platelets onto VWF, promoting platelet activation^15^. A previous study has similarly found that patients with syncope and orthostatic hypotension have increased levels of vWF during orthostasis^16^. Tubulin beta-1 (TUBB1), which is required for optimal platelet assembly, was also upregulated in POTS. Deficiency in TUBB1 leads to thrombocytopenia, whereas overexpression is associated with formation of large platelets and platelet hyperaggregation^17^.

Cartilage oligomeric matrix protein (COMP) is extracellular matrix protein is secreted by platelets and inhibits thrombin-induced platelet aggregation and activation^18^. Integrin-linked kinase (ILK) facilitates rapid platelet activation and is essential for the formation of stable thrombi^19^, and beta-parvin (PARVB), is known to play a regulatory role in integrin-signaling via ILK. Our study demonstrates that COMP was significantly downregulated in POTS, which may result in defective hemostasis and thrombosis. Similarly, ILK and PARVB were upregulated in POTS, enhancing platelet aggregation and thrombus formation.

There is limited data regarding specific complications arising from POTS and possible rates of thromboembolism^20^. As such, interventions targeting thrombogenicity in POTS, e.g., thromboprophylaxis with low-molecular-weight heparin, merit further investigation focusing on proteins associated with the von Willebrand complex, such as the involvement of GP1B.

### Cardiac contractility and hypertrophy in POTS

In addition to its prothrombic function, ILK together with PARVB also form a critical complex responsible for regulation of cardiac contractility^21^. ILK regulates diverse signal transduction pathways implicated in cardiac hypertrophy and contractility^22^. We noted that both ILK and PARVB were upregulated in POTS. To date, few studies have thoroughly assessed the cardiac morphology by echocardiography in POTS with only one small study in 19 patients have shown that individuals with POTS exhibited smaller left ventricular mass compared with 16 healthy controls^23^. Larger case–control studies with echocardiographic evaluation in POTS are warranted to elucidate whether these patients exhibit possible cardiac hypertrophy.

Thrombospondin-4 (TSP-4) is involved in regulation and remodeling of the cardiac extracellular matrix and myocyte contractility. We observed downregulated expression of TSP-4 in POTS. In vivo mouse models have shown that TSP-4 deficient mice develop pronounced cardiac hypertrophy, fibrosis together with left ventricular dilatation, and depressed systolic function^24^. These defects in adaptation to chronic pressure overload, result in chamber dilation, reduced cardiac function, and increased cardiac mass^24^.

### Proinflammatory state in POTS

Notably, the most upregulated plasma protein within the proinflammatory pathway in POTS was beta-2-microglobulin (B2M), which is a component of the major histocompatibility complex class I. Ongoing inflammation is associated with elevated levels of β2M^25^, likewise, increased levels of B2M have been reported in several autoimmune diseases. Previous studies have also found that B2M-specific autoantibodies cause platelet aggregation^26^, thereby facilitating thrombosis and providing a link between thrombogenicity and inflammation. Downregulation of WD repeat-containing protein 1 (WDR1) can cause autoinflammation and thrombocytopenia^27^, in this study, we observed enhanced expression of WDR1 in POTS.

Growing evidence points toward POTS being an autoimmune disease, oftentimes triggered by a viral or bacterial infection^28^. Lumican (LUM) interacts with CD14 and CD18 on macrophage and neutrophils to promote innate immune response, and normally helps to restrict autoimmunity, antiviral, bacterial and inflammatory responses^29^. We noted downregulated expression of LUM in POTS and hypothesized that it may result in an impaired anti-inflammatory response, unable to inhibit viral replication in these patients.

A previous study tried to elucidate the inflammatory proteomic signature of POTS using antibody-based Proximity Extension Assay (PEA) technique in nearly 400 patients, measuring simultaneously 57 inflammatory protein biomarkers^30^. Only one plasma protein, proconvertase furin, was found to be downregulated compared with individuals without POTS. Unlike our current study, the previous one did not include healthy controls, but merely individuals with a normal orthostatic response. Moreover, the proteomic methods differed between both studies. In the current study, we used a mass spectrometry-based technique, offering a more detailed molecular characterization of proteins. Rather than just detection, like the PEA, mass spectrometry can reveal unique chemical fragments of proteins, such as specific biochemical interactions^31^.

### Increased expression of skeletal muscle myosin in POTS

We observed significantly increased expression of myosin light chain 1/3, skeletal muscle isoform (MYL1) expressed only in fast skeletal muscles in adults and required for proper formation and maintenance of myofibers, and muscle function^32^. Faulty regulation in myogenesis leads to deconditioning-related changes characterized by decreased muscle mass^33^. These findings are in line with the previous identification of upregulation of myoglobin in POTS^34^. In POTS, differences in sympathetic nerve discharge and fiber loss from skeletal muscles is observed^35^, which could putatively explain muscle fatiguability and deconditioning associated with POTS.

### Upregulated alpha-adrenergic activity in POTS

We noted significant upregulation of myosin regulatory light chain 12B (MYL12B) protein in POTS, which triggers polymerization of vascular smooth muscle^36^. Vascular smooth muscle cells are predominantly innervated by the sympathetic α1 adrenergic receptors and play an important role in maintaining cardiovascular homeostasis by regulating vascular tone, blood flow and blood pressure^37^. In POTS, increased sympathetic activation causes activation of the adrenergic receptors and a surge in norepinephrine levels, and approximately 89% of patients with POTS exhibit elevated levels of autoantibodies against the adrenergic α1 receptor^38^.

### Strengths and limitations of the study

Our study is the first to use an unbiased proteomics analysis by mass spectrometry in one of the larger studies of POTS populations. To date, only a single mass spectrometry study has been conducted in POTS, with limited number of participants (10 POTS patients and seven healthy controls), which explored autoantibodies in POTS as potential novel biomarkers^39^. Here, we have thoroughly matched POTS cases with healthy controls according to age and sex, improving statistical precision and validity. Moreover, the use of mass spectrometric analysis offers several advantages compared with other methods, including high sensitivity, good proteome coverage, rapid method setup, accessibility of post-translational modifications, rendering it very attractive for unbiased biomarker discovery^40^. The recently developed DIA method achieves even higher reproducibility making technical replicates unnecessary and rendering it highly suitable for large scale population studies^7^.

Although mass spectrometry-based proteomics is an evolving technique, the sensitivity of the instruments and the high dynamic range of plasma samples does not allow detection of low-abundance proteins in plasma. The proteomic markers identified in this study ought to be replicated and validated in an independent cohort. Detection of these biomarkers does not reveal what effects they may have on target end-organs which must be the subject of further study. Since POTS is more prevalent in women than men, it is expected that the number of male patients with POTS were fewer, explaining in part why we did not observe any differences in proteomic expression between male POTS patients and healthy male controls. Moreover, the observational design of our study precludes conclusions on causality and cannot exclude unknown confounding.

### Clinical perspectives and implications

Few studies have identified promising biomarkers in POTS, and therapeutic possibilities are mainly focused on symptom relief, posing substantial challenges to physicians^41^. Our study identified novel proteomic footprints in POTS linked to increased platelet activity, proinflammatory states, and hyperadrenergic activity, facilitating a greater mechanistic understanding of the syndrome. Further investigations are warranted into the underlying molecular mechanisms leading to POTS and development of targeted therapeutic strategies.

## Conclusions

Our observations are the first to use a label-free unbiased MS-based quantification approach to elucidate the proteomic footprint of postural orthostatic tachycardia syndrome (POTS). The strongest network interaction in POTS was notably associated with a hypercoagulable state, an upregulated expression of proteins related to platelet activity. In addition, we observed proteomic patterns involved in proinflammatory states, enhanced cardiac contractility and hypertrophy, as well as adrenergic activity. These findings support the hypothesis that POTS may be an autoimmune, inflammatory and hyperadrenergic disorder.

## Supplementary Information

Supplementary Information.

## Data Availability

All authors had full access to all the data in the study and takes responsibility for its integrity and the data analysis. The mass spectrometry proteomics data have been deposited at the ProteomeXchange Consortium via the PRIDE partner repository with the dataset identifier PXD031458.

## Abbreviations

B2M: β-2-Microglobulin
COMP: Cartilage oligomeric matrix protein
DIA: Data independent acquisition
FLNA: Filamin-A
GP1BA: Platelet glycoprotein Ib alpha chain
GP1BB: Platelet glycoprotein Ib beta chain
ILK: Integrin-linked kinase
LUM: Lumican
MYL12A: Myosin regulatory light chain 12A
MYL12B: Myosin regulatory light chain 12B
PARVB: β-Parvin
POTS: Postural orthostatic tachycardia syndrome
PP1A: Peptidyl-prolyl cis–trans isomerase A
THBS4: Thrombospondin-4
TUBB1: Tubulin beta-1 chain
WDR1: WD repeat-containing protein 1
