# Supplementary material for: Plasma proteomic profiling in postural orthostatic tachycardia syndrome (POTS) reveals new disease pathways
Source: Sci Rep. 2022 Nov 21;12:20051. doi: 10.1038/s41598-022-24729-x (PMC9681882; doi:10.1038/s41598-022-24729-x)
Supplement: Supplementary file 1 — Supplementary Information. [file 41598_2022_24729_MOESM1_ESM.docx]

**Online Supplement**

***Blood Sample Preparation***

Plasma samples derived from POTS subjects and heathy controls were analyzed in a blinded manner by Bio-MS, Lund, Sweden. All samples were randomized by the biobank before being delivered to BioMS (i.e., the staff at BioMS were unaware of which patients belonged to the POTS and control group). Blood samples were collected between 2017-2020 for both POTS and controls, average time from sample collection to mass spectrometric analysis was 3 years for both groups. Sample preparation was performed on Agilent AssayMAP Bravo Platform (Agilent Technologies, Inc). Ten microliter (µL) plasma sample (diluted 1:10 in 100 mM ammonium bicarbonate) was transferred to a 96-well plate (Greiner G650201) and 40 µL of 4 M urea (Sigma-Aldrich) in 100 mM ammonium bicarbonate (Sigma-Aldrich) was manually added to a final volume of 50 µL. The Bravo platform was used for the enzyme digestion of proteins and later desalting of peptides. The proteins were reduced with 10 µL of 60 mM dithiothreitol (final concentration of 10 mM, Sigma-Aldrich) for 1 h at 37°C followed by alkylation with 20 µL of 80 mM iodoacetamide (final concentration of 20 mM, Sigma-Aldrich) for 30 min in the dark at room temperature. The plasma samples were first digested with Lys-C (FUJIFILM Wako Chemicals, USA, Corp.) at an enzyme: protein ratio of 1:50 w/w for 4 h at room temperature and further digested with trypsin (sequencing grade modified, Promega) at a trypsin: protein ratio of 1:50 w/w overnight at room temperature.

The digestion was stopped by pipetting 20 µL of 10% trifluoroacetic acid (TFA, Sigma-Aldrich) and the digested peptides were desalted on the Bravo platform. Ninety percent acetonitrile (ACN, Sigma-Aldrich) with 0.1% TFA and 0.1% TFA were used to prime and equilibrate the AssayMAP C18 cartridges (Agilent, PN: 5190-6532), respectively. The samples were loaded into the cartridges at the flow rate of 5 µL/min. The cartridges were washed with 0.1% TFA before the peptides were eluted with 80% ACN/0.1% TFA. The eluted peptides were dried in a vacuum concentrator (Eppendorf, Germany) and resuspended in 25 μL of 2% ACN/0.1% TFA. The peptide concentration was measured using Nanodrop (DeNovix Inc., Wilmington, USA). The samples were diluted to 1 µg/µL with 0.1% formic acid, spiked with synthetic iRT peptides (JPT Peptide Technologies, Germany) with the ratio of sample and iRT at 10:1 and further diluted with 0.1% formic acid to 25 ng/µL. Twenty µL of each sample was loaded into Evosep tip for mass spectrometry analysis.

**Data-independent-acquisition (DIA) data analysis**

Data-independent-acquisition (DIA) data were analyzed by using Spectronaut (version 15, Biognosys, Switzerland) with the directDIA workflow. The data was extracted based on maximum intensity for both precursors and fragment ions. The default settings were applied for the peptides and proteins identification and quantification.

In brief, Q value of 0.01 was used to estimate false discovery rate (FDRs) for both precursors and proteins identification and the p-value was calculated by kernel-density estimator. For the quantification, interference correction was activated and a minimum of 3 fragment ions and 2 precursor ions were kept. The peak area of MS2 level was used for quantitation. Peptide (stripped sequence) quantity was measured by the mean of 1–3 most intensive precursors, and protein quantity was calculated accordingly by the mean of 1–3 best peptides.

Data was filtered by q-value and automatic strategy was used for the cross-run normalization. The protein Quant report was exported from Spectronaut and downstream statistical analysis was performed with Rstudio.

Label-free quantification (LFQ) DIA data was log2-transformed and normalized to median value of each sample. If more than 30% of values in at least one group are missing values, the proteins were discarded. The missing values were replaced from the protein intensity distribution. Student’s t-test was performed with the permutation-based FDR of 0.05.

**Table S1. Complete list of 393 proteins identified using data-independent acquisition (DIA) label-free quantification mass spectrometry.**

| **Protein descriptions** | **UniProt IDs** |
| --- | --- |
| 14-3-3 protein zeta/delta | P63104 |
| 60 kDa heat shock protein, mitochondrial | P10809 |
| Actin, aortic smooth muscle;Actin, gamma-enteric smooth muscle | P62736;P63267 |
| Actin, cytoplasmic 1 | P60709 |
| Actin, cytoplasmic 2 | P63261 |
| Adenylyl cyclase-associated protein 1 | Q01518 |
| Adiponectin | Q15848 |
| Afamin | P43652 |
| Alpha-1-acid glycoprotein 1 | P02763 |
| Alpha-1-acid glycoprotein 2 | P19652 |
| Alpha-1-antichymotrypsin | P01011 |
| Alpha-1-antitrypsin | P01009 |
| Alpha-1B-glycoprotein | P04217 |
| Alpha-2-antiplasmin | P08697 |
| Alpha-2-HS-glycoprotein | P02765 |
| Alpha-2-macroglobulin | P01023 |
| Alpha-actinin-1 | P12814 |
| Alpha-enolase | P06733 |
| Aminopeptidase N | P15144 |
| Angiotensinogen | P01019 |
| Antithrombin-III | P01008 |
| Apolipoprotein A-I | P02647 |
| Apolipoprotein A-II | P02652 |
| Apolipoprotein A-IV | P06727 |
| Apolipoprotein B-100 | P04114 |
| Apolipoprotein C-I | P02654 |
| Apolipoprotein C-II | P02655 |
| Apolipoprotein C-III | P02656 |
| Apolipoprotein C-IV | P55056 |
| Apolipoprotein D | P05090 |
| Apolipoprotein E | P02649 |
| Apolipoprotein F | Q13790 |
| Apolipoprotein L1 | O14791 |
| Apolipoprotein M | O95445 |
| Apolipoprotein(a) | P08519 |
| ATP synthase subunit alpha, mitochondrial | P25705 |
| Attractin | O75882 |
| Beta-2-glycoprotein 1 | P02749 |
| Beta-2-microglobulin | P61769 |
| Beta-Ala-His dipeptidase | Q96KN2 |
| Beta-parvin | Q9HBI1 |
| Biotinidase | P43251 |
| Bleomycin hydrolase | Q13867 |
| Bone marrow proteoglycan | P13727 |
| C4b-binding protein alpha chain | P04003 |
| C4b-binding protein beta chain | P20851 |
| Cadherin-5 | P33151 |
| Calmodulin-1;Calmodulin-2;Calmodulin-3 | P0DP23;P0DP24;P0DP25 |
| Calreticulin | P27797 |
| Carbonic anhydrase 1 | P00915 |
| Carboxypeptidase B2 | Q96IY4 |
| Carboxypeptidase N catalytic chain | P15169 |
| Carboxypeptidase N subunit 2 | P22792 |
| Cartilage acidic protein 1 | Q9NQ79 |
| Cartilage oligomeric matrix protein | P49747 |
| Caveolae-associated protein 2 | O95810 |
| CD44 antigen | P16070 |
| CD5 antigen-like | O43866 |
| CD9 antigen | P21926 |
| Ceruloplasmin | P00450 |
| Chloride intracellular channel protein 1 | O00299 |
| Cholesteryl ester transfer protein | P11597 |
| Cholinesterase | P06276 |
| Clusterin | P10909 |
| Coagulation factor IX | P00740 |
| Coagulation factor V | P12259 |
| Coagulation factor VII | P08709 |
| Coagulation factor X | P00742 |
| Coagulation factor XI | P03951 |
| Coagulation factor XII | P00748 |
| Coagulation factor XIII A chain | P00488 |
| Coagulation factor XIII B chain | P05160 |
| Cofilin-1 | P23528 |
| Collectin-10 | Q9Y6Z7 |
| Collectin-11 | Q9BWP8 |
| Complement C1q subcomponent subunit A | P02745 |
| Complement C1q subcomponent subunit B | P02746 |
| Complement C1q subcomponent subunit C | P02747 |
| Complement C1r subcomponent | P00736 |
| Complement C1r subcomponent-like protein | Q9NZP8 |
| Complement C1s subcomponent | P09871 |
| Complement C2 | P06681 |
| Complement C3 | P01024 |
| Complement C4-A | P0C0L4 |
| Complement C4-B | P0C0L5 |
| Complement C5 | P01031 |
| Complement component C6 | P13671 |
| Complement component C7 | P10643 |
| Complement component C8 alpha chain | P07357 |
| Complement component C8 beta chain | P07358 |
| Complement component C8 gamma chain | P07360 |
| Complement component C9 | P02748 |
| Complement factor B | P00751 |
| Complement factor D | P00746 |
| Complement factor H | P08603 |
| Complement factor H-related protein 1 | Q03591 |
| Complement factor H-related protein 2 | P36980 |
| Complement factor H-related protein 3 | Q02985 |
| Complement factor H-related protein 4 | Q92496 |
| Complement factor I | P05156 |
| Corticosteroid-binding globulin | P08185 |
| C-reactive protein | P02741 |
| Creatine kinase M-type | P06732 |
| Cystatin-C | P01034 |
| Cysteine-rich secretory protein 3 | P54108 |
| Di-N-acetylchitobiase | Q01459 |
| Dopamine beta-hydroxylase | P09172 |
| EGF-containing fibulin-like extracellular matrix protein 1 | Q12805 |
| Endoplasmic reticulum chaperone BiP | P11021 |
| Endothelial protein C receptor | Q9UNN8 |
| Erythrocyte band 7 integral membrane protein | P27105 |
| Extracellular matrix protein 1 | Q16610 |
| Extracellular superoxide dismutase [Cu-Zn] | P08294 |
| Fat storage-inducing transmembrane protein 1 | A5D6W6 |
| Fermitin family homolog 3 | Q86UX7 |
| Fetuin-B | Q9UGM5 |
| Fibrinogen alpha chain | P02671 |
| Fibrinogen beta chain | P02675 |
| Fibrinogen gamma chain | P02679 |
| Fibronectin | P02751 |
| Fibulin-1 | P23142 |
| Ficolin-2 | Q15485 |
| Ficolin-3 | O75636 |
| Filamin-A | P21333 |
| Fructose-bisphosphate aldolase A | P04075 |
| Fructose-bisphosphate aldolase B | P05062 |
| Galectin-3-binding protein | Q08380 |
| Gamma-glutamyl hydrolase | Q92820 |
| Gelsolin | P06396 |
| Glutathione peroxidase 3 | P22352 |
| Glyceraldehyde-3-phosphate dehydrogenase | P04406 |
| Haptoglobin | P00738 |
| Haptoglobin-related protein | P00739 |
| Heat shock cognate 71 kDa protein | P11142 |
| Heat shock protein beta-1 | P04792 |
| Hemoglobin subunit alpha | P69905 |
| Hemoglobin subunit beta | P68871 |
| Hemoglobin subunit delta | P02042 |
| Hemopexin | P02790 |
| Heparin cofactor 2 | P05546 |
| Hepatocyte growth factor activator | Q04756 |
| Hepatocyte growth factor-like protein | P26927 |
| Heterogeneous nuclear ribonucleoprotein M | P52272 |
| Histidine-rich glycoprotein | P04196 |
| Histone H4 | P62805 |
| Hyaluronan-binding protein 2 | Q14520 |
| IgGFc-binding protein | Q9Y6R7 |
| Immunoglobulin alpha-2 heavy chain | P0DOX2 |
| Immunoglobulin delta heavy chain | P0DOX3 |
| Immunoglobulin gamma-1 heavy chain | P0DOX5 |
| Immunoglobulin heavy constant alpha 1 | P01876 |
| Immunoglobulin heavy constant delta | P01880 |
| Immunoglobulin heavy constant gamma 2 | P01859 |
| Immunoglobulin heavy constant gamma 3 | P01860 |
| Immunoglobulin heavy constant gamma 4 | P01861 |
| Immunoglobulin heavy constant mu | P01871 |
| Immunoglobulin heavy variable 1-18 | A0A0C4DH31 |
| Immunoglobulin heavy variable 1-2 | P23083 |
| Immunoglobulin heavy variable 1-24 | A0A0C4DH33 |
| Immunoglobulin heavy variable 1-3 | A0A0C4DH29 |
| Immunoglobulin heavy variable 1-45 | A0A0A0MS14 |
| Immunoglobulin heavy variable 1-46 | P01743 |
| Immunoglobulin heavy variable 1-58 | A0A0C4DH39 |
| Immunoglobulin heavy variable 1-69 | P01742 |
| Immunoglobulin heavy variable 1-69D | A0A0B4J2H0 |
| Immunoglobulin heavy variable 1-8 | P0DP01 |
| Immunoglobulin heavy variable 2-26 | A0A0B4J1V2 |
| Immunoglobulin heavy variable 2-5 | P01817 |
| Immunoglobulin heavy variable 2-70D | A0A0C4DH43 |
| Immunoglobulin heavy variable 3-11 | P01762 |
| Immunoglobulin heavy variable 3-13 | P01766 |
| Immunoglobulin heavy variable 3-15 | A0A0B4J1V0 |
| Immunoglobulin heavy variable 3-20 | A0A0C4DH32 |
| Immunoglobulin heavy variable 3-21 | A0A0B4J1V1 |
| Immunoglobulin heavy variable 3-23 | P01764 |
| Immunoglobulin heavy variable 3-33 | P01772 |
| Immunoglobulin heavy variable 3-43 | A0A0B4J1X8 |
| Immunoglobulin heavy variable 3-49 | A0A0A0MS15 |
| Immunoglobulin heavy variable 3-53 | P01767 |
| Immunoglobulin heavy variable 3-64 | A0A075B6Q5 |
| Immunoglobulin heavy variable 3-64D | A0A0J9YX35 |
| Immunoglobulin heavy variable 3-7 | P01780 |
| Immunoglobulin heavy variable 3-72 | A0A0B4J1Y9 |
| Immunoglobulin heavy variable 3-73 | A0A0B4J1V6 |
| Immunoglobulin heavy variable 3-74 | A0A0B4J1X5 |
| Immunoglobulin heavy variable 3-9 | P01782 |
| Immunoglobulin heavy variable 4-28 | A0A0C4DH34 |
| Immunoglobulin heavy variable 4-34 | P06331 |
| Immunoglobulin heavy variable 4-38-2 | P0DP08 |
| Immunoglobulin heavy variable 4-4 | A0A075B6R2 |
| Immunoglobulin heavy variable 5-10-1 | A0A0J9YXX1 |
| Immunoglobulin heavy variable 5-51 | A0A0C4DH38 |
| Immunoglobulin heavy variable 6-1 | A0A0B4J1U7 |
| Immunoglobulin J chain | P01591 |
| Immunoglobulin kappa constant | P01834 |
| Immunoglobulin kappa light chain | P0DOX7 |
| Immunoglobulin kappa variable 1-12;Immunoglobulin kappa variable 1D-12 | A0A0C4DH73;P01611 |
| Immunoglobulin kappa variable 1-16 | P04430 |
| Immunoglobulin kappa variable 1-17 | P01599 |
| Immunoglobulin kappa variable 1-27 | A0A075B6S5 |
| Immunoglobulin kappa variable 1-39;Immunoglobulin kappa variable 1D-39 | P01597;P04432 |
| Immunoglobulin kappa variable 1-5 | P01602 |
| Immunoglobulin kappa variable 1-8 | A0A0C4DH67 |
| Immunoglobulin kappa variable 1D-13;Immunoglobulin kappa variable 1-13 | A0A0B4J2D9;P0DP09 |
| Immunoglobulin kappa variable 1D-16 | P01601 |
| Immunoglobulin kappa variable 1D-33;Immunoglobulin kappa variable 1-33 | P01593;P01594 |
| Immunoglobulin kappa variable 1D-43 | A0A0B4J1Z2 |
| Immunoglobulin kappa variable 1D-8 | A0A087WSZ0 |
| Immunoglobulin kappa variable 2-28;Immunoglobulin kappa variable 2D-28 | A0A075B6P5;P01615 |
| Immunoglobulin kappa variable 2-29 | A2NJV5 |
| Immunoglobulin kappa variable 2-40;Immunoglobulin kappa variable 2D-40 | A0A087WW87;P01614 |
| Immunoglobulin kappa variable 2D-29 | A0A075B6S2 |
| Immunoglobulin kappa variable 2D-30;Immunoglobulin kappa variable 2-30 | A0A075B6S6;P06310 |
| Immunoglobulin kappa variable 3-11 | P04433 |
| Immunoglobulin kappa variable 3-15 | P01624 |
| Immunoglobulin kappa variable 3-20 | P01619 |
| Immunoglobulin kappa variable 3D-15 | A0A087WSY6 |
| Immunoglobulin kappa variable 3D-20 | A0A0C4DH25 |
| Immunoglobulin kappa variable 3D-7 | A0A0C4DH55 |
| Immunoglobulin kappa variable 4-1 | P06312 |
| Immunoglobulin kappa variable 5-2 | P06315 |
| Immunoglobulin kappa variable 6-21 | A0A0C4DH24 |
| Immunoglobulin kappa variable 6D-21 | A0A0A0MT36 |
| Immunoglobulin lambda constant 2;Immunoglobulin lambda constant 3 | P0DOY2;P0DOY3 |
| Immunoglobulin lambda variable 1-36 | A0A0B4J1U3 |
| Immunoglobulin lambda variable 1-40 | P01703 |
| Immunoglobulin lambda variable 1-44 | P01699 |
| Immunoglobulin lambda variable 1-47 | P01700 |
| Immunoglobulin lambda variable 1-51 | P01701 |
| Immunoglobulin lambda variable 2-11 | P01706 |
| Immunoglobulin lambda variable 2-14 | P01704 |
| Immunoglobulin lambda variable 2-18 | A0A075B6J9 |
| Immunoglobulin lambda variable 2-23 | P01705 |
| Immunoglobulin lambda variable 3-1 | P01715 |
| Immunoglobulin lambda variable 3-10 | A0A075B6K4 |
| Immunoglobulin lambda variable 3-19 | P01714 |
| Immunoglobulin lambda variable 3-21 | P80748 |
| Immunoglobulin lambda variable 3-25 | P01717 |
| Immunoglobulin lambda variable 3-9 | A0A075B6K5 |
| Immunoglobulin lambda variable 5-37 | A0A075B6J1 |
| Immunoglobulin lambda variable 5-39 | A0A0G2JS06 |
| Immunoglobulin lambda variable 6-57 | P01721 |
| Immunoglobulin lambda variable 7-43 | P04211 |
| Immunoglobulin lambda variable 8-61 | A0A075B6I0 |
| Immunoglobulin lambda variable 9-49 | A0A0B4J1Y8 |
| Immunoglobulin lambda-like polypeptide 1 | P15814 |
| Immunoglobulin lambda-like polypeptide 5;Immunoglobulin lambda-1 light chain | B9A064;P0DOX8 |
| Immunoglobulin mu heavy chain | P0DOX6 |
| Insulin-like growth factor II | P01344 |
| Insulin-like growth factor-binding protein 3 | P17936 |
| Insulin-like growth factor-binding protein complex acid labile subunit | P35858 |
| Integrin alpha-IIb | P08514 |
| Integrin beta-3 | P05106 |
| Integrin-linked protein kinase | Q13418 |
| Inter-alpha-trypsin inhibitor heavy chain H1 | P19827 |
| Inter-alpha-trypsin inhibitor heavy chain H2 | P19823 |
| Inter-alpha-trypsin inhibitor heavy chain H3 | Q06033 |
| Inter-alpha-trypsin inhibitor heavy chain H4 | Q14624 |
| Intercellular adhesion molecule 2 | P13598 |
| Interleukin-1 receptor accessory protein | Q9NPH3 |
| Kallistatin | P29622 |
| Keratin, type I cytoskeletal 10 | P13645 |
| Keratin, type I cytoskeletal 14 | P02533 |
| Keratin, type I cytoskeletal 16 | P08779 |
| Keratin, type I cytoskeletal 17 | Q04695 |
| Keratin, type I cytoskeletal 9 | P35527 |
| Keratin, type II cytoskeletal 1 | P04264 |
| Keratin, type II cytoskeletal 2 epidermal | P35908 |
| Keratin, type II cytoskeletal 5 | P13647 |
| Keratin, type II cytoskeletal 6A | P02538 |
| Keratin, type II cytoskeletal 6B | P04259 |
| Kininogen-1 | P01042 |
| Leucine-rich alpha-2-glycoprotein | P02750 |
| LIM and senescent cell antigen-like-containing domain protein 1 | P48059 |
| Lipopolysaccharide-binding protein | P18428 |
| L-lactate dehydrogenase A chain | P00338 |
| L-lactate dehydrogenase B chain | P07195 |
| Low affinity immunoglobulin gamma Fc region receptor III-A | P08637 |
| L-selectin | P14151 |
| Lumican | P51884 |
| Lymphatic vessel endothelial hyaluronic acid receptor 1 | Q9Y5Y7 |
| Lysozyme C | P61626 |
| Mannan-binding lectin serine protease 1 | P48740 |
| Mannose-binding protein C | P11226 |
| Monocyte differentiation antigen CD14 | P08571 |
| Multimerin-1 | Q13201 |
| Multimerin-2 | Q9H8L6 |
| Myosin light chain 1/3, skeletal muscle isoform | P05976 |
| Myosin light polypeptide 6 | P60660 |
| Myosin regulatory light chain 12B;Myosin regulatory light chain 12A | O14950;P19105 |
| Myosin regulatory light polypeptide 9 | P24844 |
| Myosin-9 | P35579 |
| N-acetylmuramoyl-L-alanine amidase | Q96PD5 |
| Nucleophosmin | P06748 |
| Pantetheinase;Vascular non-inflammatory molecule 3 | O95497;Q9NY84 |
| PDZ and LIM domain protein 1 | O00151 |
| Peptidase inhibitor 16 | Q6UXB8 |
| Peptidyl-prolyl cis-trans isomerase A | P62937 |
| Phosphatidylcholine-sterol acyltransferase | P04180 |
| Phosphatidylinositol-glycan-specific phospholipase D | P80108 |
| Phosphoglycerate kinase 1 | P00558 |
| Phospholipid transfer protein | P55058 |
| Pigment epithelium-derived factor | P36955 |
| Plasma kallikrein | P03952 |
| Plasma protease C1 inhibitor | P05155 |
| Plasma serine protease inhibitor | P05154 |
| Plasminogen | P00747 |
| Platelet basic protein | P02775 |
| Platelet factor 4 | P02776 |
| Platelet glycoprotein 4 | P16671 |
| Platelet glycoprotein Ib alpha chain | P07359 |
| Platelet glycoprotein Ib beta chain | P13224 |
| Platelet glycoprotein V | P40197 |
| Pleckstrin | P08567 |
| Polymeric immunoglobulin receptor | P01833 |
| Polyubiquitin-B;Polyubiquitin-C;Ubiquitin-40S ribosomal protein S27a;Ubiquitin-60S ribosomal protein L40 | P0CG47;P0CG48;P62979;P62987 |
| Pregnancy zone protein | P20742 |
| Prenylcysteine oxidase 1 | Q9UHG3 |
| Probable non-functional immunoglobulin heavy variable 3-35 | A0A0C4DH35 |
| Probable non-functional immunoglobulin heavy variable 3-38 | A0A0C4DH36 |
| Probable non-functional immunoglobulin kappa variable 2D-24;Immunoglobulin kappa variable 2-24 | A0A075B6R9;A0A0C4DH68 |
| Probable non-functional immunoglobulin kappa variable 3-7 | A0A075B6H7 |
| Probable non-functional immunoglobulin lambda variable 1-50 | A0A075B6I6 |
| Probable non-functional immunoglobulin lambda variable 5-48 | A0A075B6I7 |
| Probable non-functional immunoglobulinn kappa variable 1-37;Probable non-functional immunoglobulinn kappa variable 1D-37 | A0A075B6S9;P0DSN7 |
| Procollagen C-endopeptidase enhancer 1 | Q15113 |
| Profilin-1 | P07737 |
| Properdin | P27918 |
| Prostaglandin-H2 D-isomerase | P41222 |
| Protein AMBP | P02760 |
| Protein disulfide-isomerase A3 | P30101 |
| Protein KIAA0100 | Q14667 |
| Protein S100-A9 | P06702 |
| Protein Z-dependent protease inhibitor | Q9UK55 |
| Proteoglycan 4 | Q92954 |
| Prothrombin | P00734 |
| Prothymosin alpha | P06454 |
| Putative HLA class I histocompatibility antigen, alpha chain H;HLA class I histocompatibility antigen, A alpha chain | P01893;P04439 |
| Pyruvate kinase PKM | P14618 |
| Ras-related protein Rap-1b | P61224 |
| Retinol-binding protein 4 | P02753 |
| Secreted phosphoprotein 24 | Q13103 |
| Selenoprotein P | P49908 |
| Serglycin | P10124 |
| Serine hydrolase RBBP9 | O75884 |
| Serine/threonine-protein kinase PAK 6 | Q9NQU5 |
| Serotransferrin | P02787 |
| Serpin B12 | Q96P63 |
| Serpin B3;Serpin B4 | P29508;P48594 |
| Serum albumin | P02768 |
| Serum amyloid A-1 protein | P0DJI8 |
| Serum amyloid A-2 protein | P0DJI9 |
| Serum amyloid A-4 protein | P35542 |
| Serum amyloid P-component | P02743 |
| Serum paraoxonase/arylesterase 1 | P27169 |
| Serum paraoxonase/lactonase 3 | Q15166 |
| Sex hormone-binding globulin | P04278 |
| SH3 domain-binding glutamic acid-rich-like protein 3 | Q9H299 |
| Sulfhydryl oxidase 1 | O00391 |
| Talin-1 | Q9Y490 |
| Tenascin-X | P22105 |
| Tetranectin | P05452 |
| Thrombospondin-1 | P07996 |
| Thrombospondin-4 | P35443 |
| Thymosin beta-4 | P62328 |
| Thyroxine-binding globulin | P05543 |
| Transferrin receptor protein 1 | P02786 |
| Transforming growth factor-beta-induced protein ig-h3 | Q15582 |
| Transgelin-2 | P37802 |
| Transmembrane protein KIAA1109 | Q2LD37 |
| Transthyretin | P02766 |
| Tropomyosin alpha-1 chain | P09493 |
| Tropomyosin alpha-3 chain | P06753 |
| Tropomyosin alpha-4 chain | P67936 |
| Trypsin-1;Trypsin-2;Putative trypsin-6 | P07477;P07478;Q8NHM4 |
| Trypsin-3 | P35030 |
| Tubulin alpha-1B chain | P68363 |
| Tubulin alpha-4A chain | P68366 |
| Tubulin beta chain;Tubulin beta-4B chain | P07437;P68371 |
| Tubulin beta-1 chain | Q9H4B7 |
| Vasorin | Q6EMK4 |
| Vinculin | P18206 |
| Vitamin D-binding protein | P02774 |
| Vitamin K-dependent protein C | P04070 |
| Vitamin K-dependent protein S | P07225 |
| Vitamin K-dependent protein Z | P22891 |
| Vitronectin | P04004 |
| von Willebrand factor | P04275 |
| von Willebrand factor A domain-containing protein 8 | A3KMH1 |
| WD repeat-containing protein 1 | O75083 |
| WD repeat-containing protein WRAP73 | Q9P2S5 |
| Zinc-alpha-2-glycoprotein | P25311 |
| Zyxin | Q15942 |

**Table S2. STRING Pathway Enrichment Analysis of Up-regulated Proteins in POTS.**

| **Term ID** | **Description** | **FDR** | **Observed gene count** | **Background gene count** | **Proteins** |
| --- | --- | --- | --- | --- | --- |
| GO:0070527 | Platelet aggregation | 6.88e-06 | 5 | 42 | MYL12A,ITGA2B,GP1BA,FLNA,ILK |
| GO:0030168 | Platelet activation | 1.39e-05 | 6 | 135 | MYL12A,ITGA2B,GP1BA,FLNA,ILK,GP1BB |
| GO:0007155 | Cell adhesion | 0.0013 | 8 | 925 | MYL12A,ITGA2B,GP1BA,FLNA,ACTN1,ILK,GP1BB,PARVB |
| GO:0001775 | Cell activation | 0.0029 | 8 | 1075 | MYL12A,ITGA2B,GP1BA,FLNA,ILK,GP1BB,PPIA,B2M |
| GO:0002576 | Platelet degranulation | 0.0062 | 4 | 129 | ITGA2B,FLNA,ACTN1,WDR1 |
| GO:0031589 | Cell-substrate adhesion | 0.0199 | 4 | 182 | ITGA2B,ACTN1,ILK,PARVB |
| GO:0045055 | Regulated exocytosis | 0.0230 | 6 | 697 | ITGA2B,FLNA,ACTN1,PPIA,WDR1,B2M |
| GO:0000902 | Cell morphogenesis | 0.0271 | 6 | 726 | GP1BA,FLNA,ACTN1,ILK,PARVB,WDR1 |
| GO:0097435 | Supramolecular fiber organization | 0.0427 | 5 | 480 | FLNA,ACTN1,ILK,WDR1,B2M |
| GO:0022604 | Regulation of cell morphogenesis | 0.0481 | 5 | 498 | FLNA,ILK,PARVB,WDR1,MYL12B |

**Table legend:**

FDR (False Discovery Rate) describes how significant the protein enrichment is, and values are shown as p-values corrected for multiple testing within each category using the Benjamini–Hochberg procedure.

Observed gene count indicates how many proteins were annotated with a particular term.

Background gene count indicates how many proteins in total have this term assigned.

**Figure S1. Protein-protein pathway analysis illustrating significantly upregulated proteins in POTS with a p-value <0.05.**

**
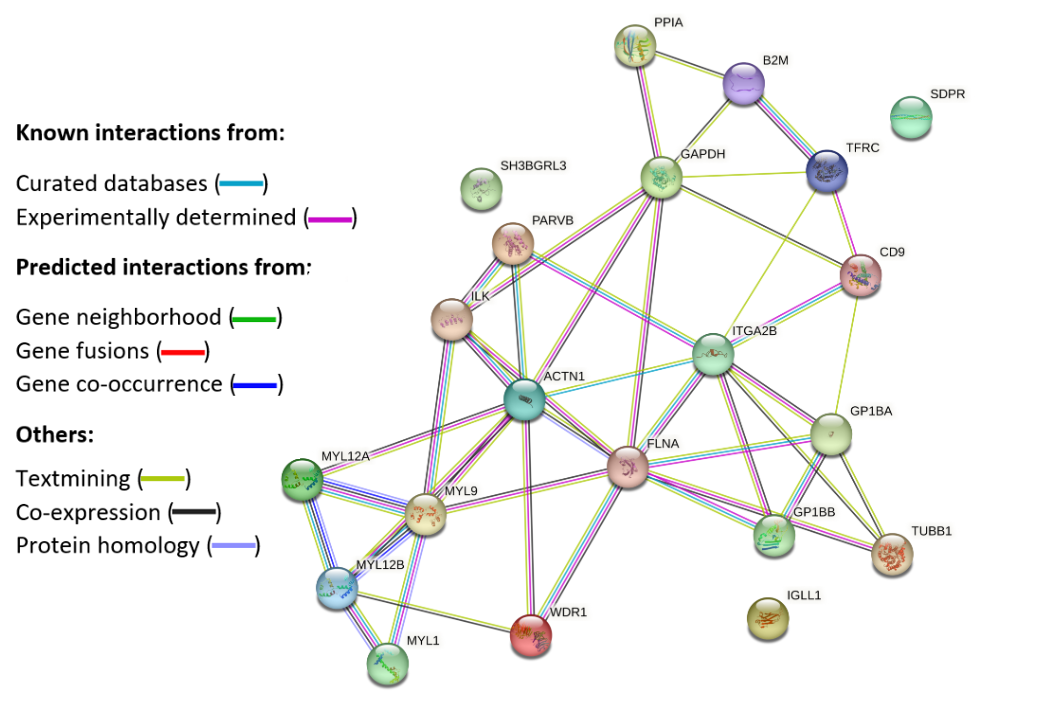
**

Protein-protein interaction analysis of all 21 significantly upregulated proteins with only a p-value <0.05 with association networks detected by STRING in POTS. In the network figures, each protein is represented by a colored node while protein–protein interaction and association are represented by a line. The strongest network interactions in POTS were particularly associated with a hypercoagulable state and upregulated expression of proteins related to platelet activity, but also enhanced inflammation, cardiac contractility and hypertrophy, skeletal muscle expression, and adrenergic activity. Complete names of proteins are found in Supplementary table S1.
